# Put your money where your feet are: The real-world effects of StepBet gamified deposit contracts for physical activity

**DOI:** 10.1016/j.invent.2023.100610

**Published:** 2023-02-26

**Authors:** David R. de Buisonjé, Fiona Brosig, Linda D. Breeman, Erika Litvin Bloom, Thomas Reijnders, Veronica R. Janssen, Roderik A. Kraaijenhagen, Hareld M.C. Kemps, Andrea W.M. Evers

**Affiliations:** a Health, Medical and Neuropsychology Unit, Institute of Psychology, Leiden University, Leiden, the Netherlands; b WayBetter, Inc., Wilmington, DE, USA; c Department of Human-Centered Design, Faculty of Industrial Design Engineering, TU Delft, Delft, the Netherlands; d Department of Cardiology, Leiden University Medical Center, Leiden, the Netherlands; e Hearts4People foundation, Amsterdam, the Netherlands; f Department of Cardiology, Máxima Medical Center, Veldhoven, the Netherlands; g Department of Industrial Design, Eindhoven University of Technology, Eindhoven, the Netherlands; h Medical Delta, Leiden University, TU Delft, and Erasmus University, the Netherlands

**Keywords:** Financial incentives, Deposit contracts, Gamification, Physical activity, Health behavior change

## Abstract

**Background:**

Gamification and deposit contracts (a financial incentive in which participants pledge their own money) can enhance effectiveness of mobile behavior change interventions. However, to assess their potential for improving population health, research should investigate implementation of gamified deposit contracts outside the research setting. Therefore, we analyzed data from StepBet, a smartphone application originally developed by WayBetter, Inc.

**Objective:**

To perform a naturalistic evaluation of StepBet gamified deposit contracts, for whom they work best, and under which conditions they are most effective to help increase physical activity.

**Methods:**

WayBetter provided data of StepBet participants that participated in a stepcount challenge between 2015 and 2020 (N = 72,974). StepBet challenges were offered on the StepBet smartphone application. The modal challenge consisted of a $40 deposit made prior to a 6-week challenge period during which participants needed to reach daily and weekly step goals in order to regain their deposit. Participants who met their goals also received additional earnings which were paid out from the money lost by those who failed their challenge. Challenge step goals were tailored on a 90-day historic step count retrieval that was also used as the baseline comparison for this study. Primary outcomes were increase in step count (continuous) and challenge success (dichotomous).

**Results:**

Overall, average daily step counts increased by 31.2 % (2423 steps, *SD* = 3462) from 7774 steps *(SD =* 3112) at baseline to 10,197 steps *(SD =* 4162*)* during the challenge. The average challenge success rate was 73 %. Those who succeeded in their challenge (n = 53,281) increased their step count by 44.0 % (3465 steps, *SD* = 3013), while those who failed their challenge (n = 19,693) decreased their step count by −5.3 % (−398 steps, *SD* = 3013). Challenges started as a New Year's resolution were slightly more successful (77.7 %) than those started during the rest of the year (72.6 %).

**Discussion:**

In a real-world setting, and among a large and diverse sample, participating in a gamified deposit contract challenge was associated with a large increase in step counts. A majority of challenges were successful and succeeding in a challenge was associated with a large and clinically relevant increase in step counts. Based on these findings, we recommend implementing gamified deposit contracts for physical activity where possible. An interesting avenue for future research is to explore possible setback effects among people who fail a challenge, and how setbacks can be mitigated.

**Pre-registration:**

Open Science Framework (doi:10.17605/OSF.IO/D237C).

## Highlights

• We did a naturalistic evaluation of 72,974 StepBet gamified deposit contract challenges to increase daily step counts
• Participating in a challenge was associated with a large and clinically relevant increase in step counts
• New Year’s resolution challenges were more effective than regular challenges
• Based on these findings, we recommend implementing deposit contracts for physical activity where possible
• We urge for more research into potential setback effects (and how to mitigate them) among those who fail their challenge

## 1 Introduction

Physical inactivity is one of the key risk factors for non-communicable diseases and causes millions of preventable deaths (World Health Organization, 2009). While physical inactivity is linked to chronic disease and early death (Anderson and Durstine, 2019), increasing physical activity improves mental health, reduces chronic disease, and increases longevity (Pedersen and Saltin, 2015). Importantly, these effects are found not only for intense aerobic training, but also for the mere number of steps taken in daily life (Lee et al., 2019; Saint-Maurice et al., 2020). Due to technological advances, steps taken in daily life can now easily be measured with the sensors that are available in smartphones. Besides allowing for real-time measurement of physical activity behavior (change), smartphones offer unique intervention opportunities. Many people habitually check their smartphone every 5 minutes, from the moment they wake up until the moment they go to bed (Heitmayer and Lahlou, 2021). Therefore, mobile behavior change interventions delivered on a smartphone have important benefits over traditional interventions (Murray et al., 2016). Instead of requiring resource intensive face-to-face contact, mobile behavior change interventions can be delivered cost-effectively to a broad audience and provide on-demand support, tailored to the dynamic nature of real-life behavior change (Mair et al., 2022). Despite these benefits, mobile behavior change interventions often suffer from a lack of adherence and high levels of attrition (Short et al., 2018). A strategy that is increasingly used to enhance engagement with mobile interventions is gamification (Alahäivälä and Oinas-Kukkonen, 2016). Gamification is defined as the use of game design elements in non-game contexts (Cugelman, 2013). The idea is that certain elements of games are highly engaging and can be incorporated in behavior change interventions to make them more engaging too. Cugelman (2013) has identified 7 persuasive strategies that are commonly applied in the gamification of behavior change. These are goal setting, challenges, feedback on performance, reinforcement, comparing progress, social connectivity, and fun and playfulness. A systematic review has shown that gamification can positively impact the effectiveness of health behavior change interventions, with the strongest evidence found for improving physical activity (Johnson et al., 2016). Furthermore, a meta-analysis has shown that gamified interventions for physical activity are not only effective in changing behavior, but also more effective compared with other behavioral interventions (Mazeas et al., 2022). It appears that adding gamification elements increases engagement with and effectiveness of mobile behavior change interventions.

StepBet (“StepBet,” n.d.), a smartphone application originally developed by WayBetter, Inc., offers commercially accessible gamified mobile walking (stepcount) challenges. WayBetter also offers gamified behavior change interventions for weight loss in their DietBet (Leahey and Rosen, 2014) and WayBetter apps and is developing QuitBet for cigarette smoking cessation (Bloom et al., 2021). WayBetter proposes that the three main components of their challenges are the use of gamified microgoals, financial incentives and social support (“Waybetter,” n.d.). In Waybetter challenges, participants deposit some of their own money into a pool and join a group challenge with a concrete goal to improve their lifestyle. During the challenge they are provided with personally tailored goals, feedback on their goal progress, and they can interact with other participants to discuss and compare their progress. At the end of a challenge, those who failed lose their initial deposit while winners split the entire pool of money and receive a full refund of their deposit plus a profit. Although the Waybetter challenges contain all 7 persuasive gamification strategies identified by Cugelman (2013), a key element is the monetary ‘bet’ participants make at the start of a challenge. Theoretically, this type of financial incentive (in which participants pledge their own money as an incentive) is referred to as a deposit contract (Stedman-Falls and Dallery, 2020). The use of deposit contracts is often argued for using present bias and loss aversion (e.g., Halpern et al., 2012). Present bias is the finding that people tend to procrastinate on their long-term goals because they are more strongly influenced by the here and now (Laibson, 1997). Loss aversion refers to the finding that people are more strongly influenced by potential losses than they are by potential gains (Kahneman and Tversky, 1979). We argue that gamified deposit contracts hold promise as a tool to increase engagement with and effectiveness of mobile behavior change interventions, because people put something of themselves ‘on the line’ in the here and now, and have fun doing so. Deposit contracts have been successfully applied to weight loss (Lesser et al., 2018; Sykes-Muskett et al., 2015), smoking cessation (Halpern et al., 2015; Jarvis and Dallery, 2017) and to increase physical activity (Budworth et al., 2019; Burns and Rothman, 2018; de Buisonjé et al., 2022; Donlin Washington et al., 2016; Krebs and Nyein, 2021; Patel et al., 2016; Stedman-Falls and Dallery, 2020). Interestingly, a recent meta-analysis of different types of financial incentives has shown that deposit contracts are the most effective financial incentive for improving healthy diet, weight control and physical activity (Boonmanunt et al., 2022).

The evidence for the effectiveness of adding gamification elements and deposit contracts to mobile behavior change interventions is promising. However, to improve population health, research has to investigate implementation of gamified deposit contracts outside the research setting and among larger and more diverse samples. Mobile behavior change interventions are often developed for research purposes, tested among WEIRD (White, Educated, Industrialized, Rich, Democratic) samples (see Rad et al., 2018), and only made available for the limited duration of a research study. In contrast, the StepBet challenges provide the opportunity to perform an ecologically valid investigation into the effect of gamified deposit contract challenges. Understanding whether gamified deposit contracts are not only efficacious in research settings, but also effective in real life conditions may inform public health policy making and may inspire future intervention design. Previous scientific evaluations of the gamified deposit contracts offered by Waybetter have shown that they are effective for weight loss (Hirt-Schierbaum and Ivets, 2020; Leahey and Rosen, 2014) and acceptable for smoking cessation (Bloom et al., 2021). Interestingly, larger bet amounts, more frequent self-monitoring, more social interactions in the app, and more sharing on social media were associated with larger weight loss (Hirt-Schierbaum and Ivets, 2020; Leahey and Rosen, 2014). With regards to when challenges are started, research has shown that a ‘fresh start’ effect exists. People are more interested and committed to pursue lifestyle goals following temporal landmarks such as the passage of the year (Dai et al., 2014). Although interest in dieting and weight loss spikes right after the new year (Dai et al., 2014), it is not known whether people are also more successful in achieving goals that are started as a New Year's resolution. On the contrary, DietBet challenges for weight loss started as a New Year's resolution were less successful than challenges started during any other period of the year (Hirt-Schierbaum and Ivets, 2020). Perhaps these New Year's resolution challenges attract more naive participants, who underestimate their future self-regulation difficulties to a greater extent (Hirt-Schierbaum and Ivets, 2020). The effects of StepBet challenges on physical activity have not yet been scientifically evaluated.

### 1.1 The current study

The primary aim of this study is to perform a naturalistic evaluation of the effect of participating in a StepBet challenge with gamified deposit contracts. Furthermore, we explore for whom these challenges work best, and under which conditions they are most effective to help increase physical activity. Based on evidence with regards to gamification (Mazeas et al., 2022) and deposit contracts (Budworth et al., 2019; Burns and Rothman, 2018; de Buisonjé et al., 2022; Donlin Washington et al., 2016; Krebs and Nyein, 2021; Patel et al., 2016; Stedman-Falls and Dallery, 2020), we hypothesize that participating in a StepBet challenge is associated with an increase in step counts. Furthermore, based on previous research on weight loss (Hirt-Schierbaum and Ivets, 2020), we hypothesize that StepBet challenges started as a New Year's resolution (between the 1st and 14th of January) have lower odds of success compared to challenges started during all other periods. Finally, we explore which features of deposit contracts or demographic variables are predictive of challenge success and increases in step counts.

## 2 Method

### 2.1 Participants

**Fig. 1:**
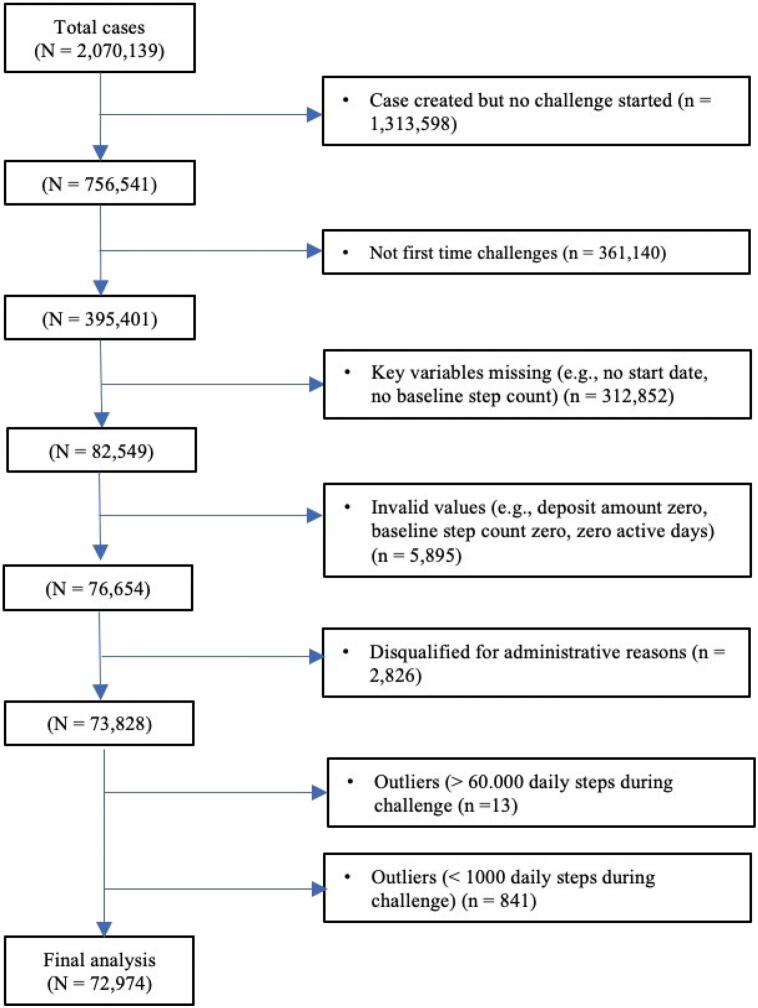
Flowchart of data cleaning process.

We analyzed data of in total 72,974 unique participants. These individuals participated in at least one StepBet challenge in the 5-year time span between 11 December 2015 and 16 March 2020. The original data file we received from WayBetter contained over 2,000,000 cases and contained all challenges that were registered on the platform during the timespan mentioned above. Prior to analysis we cleaned the dataset and excluded outlier cases with a daily average step count during the challenge of >60,000 steps per day (n = 13) or < 1000 steps per day (n = 841) (see *Appendix B* for rationale). See Fig. 1 for a flowchart of the data cleaning process. Final analysis was performed on data of 72,974 unique participants who participated in their first-time StepBet challenge. We did not obtain informed consent before the start of the study since we used anonymous research data collected by StepBet. WayBetter informs its users about the possibility of academic research on anonymized data in their privacy policy statement. The study protocol was preregistered on Open Science Framework: doi:10.17605/OSF.IO/D237C.

### 2.2 Procedure

WayBetter collected the data of StepBet participants and provided this to researchers from Leiden University. Since participants were customers of StepBet who by themselves decided to participate in these challenges, we characterize the data collection procedure as convenience sampling. To participate in a StepBet challenge, participants had to download the StepBet smartphone application (see section *The StepBet app* for more detail), allow it to record their step counts, and enter a challenge that requires a monetary deposit. During onboarding, participants were first asked to connect to their existing health tracking device (e.g., Fitbit, Garmin, Apple Health, Google Fit) for synchronization of their step count data. In doing so, the participant also allowed StepBet to retrieve their daily step count for the previous 90 days. This historic step count was used to determine a baseline and calculate a personalised step goal for during the challenge. Importantly, StepBet tried retrieving step counts for the previous 90 days, but considered 30 days as a minimum requirement and removed outlier days to calculate a baseline and tailor intervention goals (see *Appendix B* for more detail on the goal setting algorithm). Thereafter, participants were required to sign up to StepBet (via existing Social Media apps or their email account) and pick a challenge that they wanted to participate in. Upon entering a challenge, participants needed to pay the deposit amount required for the challenge (via PayPal or credit card) and then wait until it started. Most challenges started with a warm-up week during which steps were already being recorded, but participants would not fail their challenge if they did not reach their step goals. After the warm-up week the actual challenge began, and participants had to reach their daily step goal for a certain number of days per week (see section *The StepBet app* for more detail on goal setting) and received push notifications to inform them about their progress. If participants failed their challenge, they were disqualified and they lost their bet. If participants would fall ill during a challenge, they could request a refund of their deposit. When participants completed their challenge successfully (i.e., winners), they received their initial bet back plus a profit. The amount of this profit was determined by how many participants in that challenge failed and lost their deposit, such that the total amount of the deposits from failed participants was split equally among the winners. The business model of StepBet consists of both membership fees and a cut taken from challenge pots. In the unlikely event that everyone in the game is a winner (or the win rate is so high that winners would not regain their entire bet if the company took their standard cut), the company forfeits their cut.

### 2.3 Materials

#### 2.3.1 The StepBet app

**Fig. 2:**
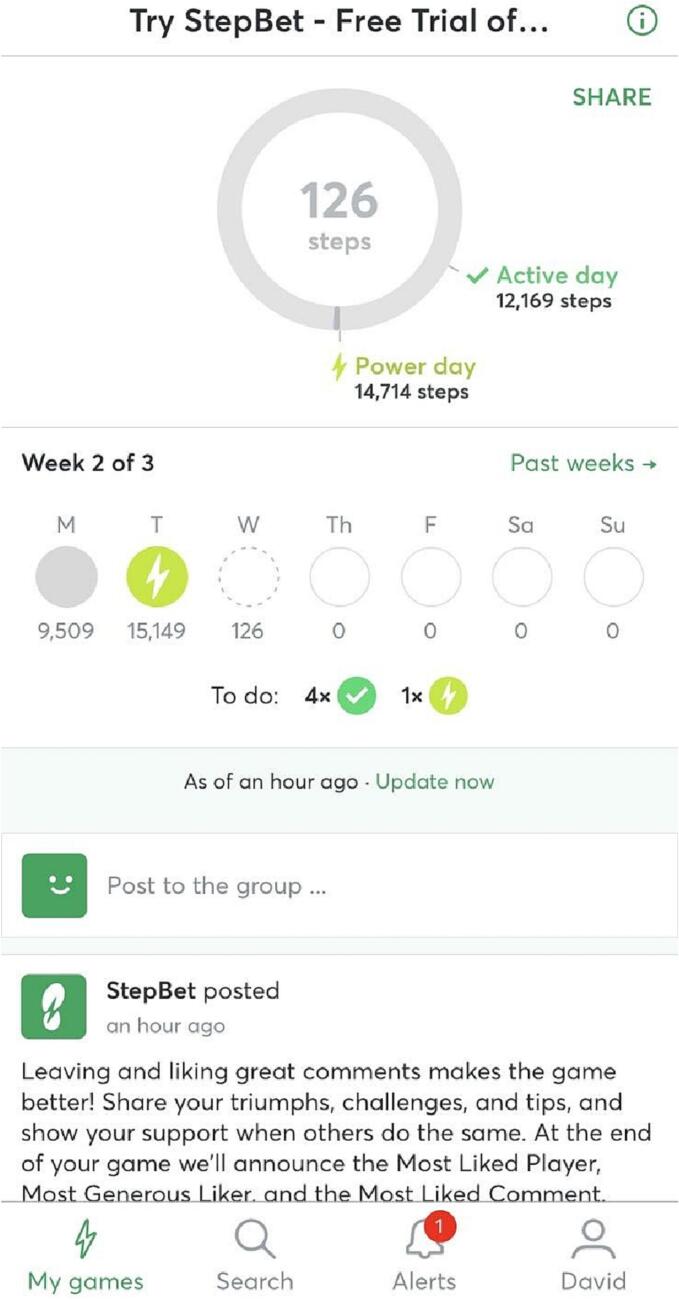
Screenshot of the StepBet application.

In the StepBet app (see Fig. 2), participants entered into a deposit contract and paid an amount of money that they could earn back by reaching daily and weekly step goals. The amount of this deposit varied per challenge and ranged between a minimum of 10$ and a maximum of 60$ (N = 72,974, *M* = 37.78 $US, *SD* = 6.79). Challenges varied in duration (N = 72,974, *M* = 40.86 days, *SD* = 2.97) but most challenges lasted 6 weeks and included a warmup week. Furthermore, challenges varied in the number of participants who participated (N = 72,974, *M* = 864.29 participants, *SD* = 797.54). Also, participants had to achieve weekly goals which were made up of a certain ratio of daily goals. The ratio of these different goal types (rest-, active-, and power days) differed per challenge, but in a modal challenge, participants had to achieve step goals on 4 active days (110 % of baseline steps), 2 power days (130 % of baseline steps) and got 1 rest day on which they didn't have to reach any certain number of steps. If the participant did not achieve the step goal on at least 4 active days and 2 power days for one week, the challenge was failed and the deposit was lost. The participant could choose on which day they reached which goal, but they needed to reach each of those goals on a weekly basis. Once a challenge was failed, participants could continue to track their steps and achieve daily and weekly goals but would no longer be able to get their deposit back. Participants could interact socially through the application by posting about their achievement, see those of others, and liking and commenting on posts of others. Participants received push notifications from the app and emails throughout the challenge. These notifications were provided to increase the frequency with which participants opened the StepBet app, to inform them about their daily goal achievement or failure, and to inform participants on whether the challenge was failed or successfully completed.

#### 2.3.2 Measures

The StepBet app automatically registered general information about challenges and challenge outcome (failure/success). Furthermore, the app automatically retrieved baseline step counts, calculated personalised goals and automatically recorded step counts during a challenge. Participants could connect an existing health tracking device (e.g., Fitbit, Garmin, Apple Watch) or use the internal gyroscope-based sensors in their smartphone to report their step counts. Algorithms recode the raw data from these sensors into an estimated step count. Most studies that investigated the validity of tracking step counts with commercially available devices (in free living conditions), showed acceptable levels of measurement error (<10 % measurement error) (Fuller et al., 2020). Overall, commercial trackers tend to slightly overestimate actual step counts, but differences exist between brands and devices (Fuller et al., 2020). The interdevice reliability of measuring step counts is overall very strong, while the intradevice reliability was found to be moderate with an average correlation coefficient between measurements of 0.58 (Fuller et al., 2020). To prevent cheating, StepBet actively monitors players' steps and flag any suspicious behavior. Players may be asked to provide additional data from their phone or tracking device if they are flagged (“StepBet FAQ,” n.d.). On a voluntary basis, participants also entered demographic information such as their birthdate, gender, and region of residence. Age was determined based on birthdate at the moment of registration in the app. Because all demographic information was provided on a voluntary basis, we do not have complete information for all participants (see Table 1 for an overview of the sample characteristics).

**Table 1:** Sample characteristics (N = 72,974).

Variable	n (%)
Sex	
Total valid	61,502
Female	53,241 (86.6 %)
Male	8261 (13.4 %)
Region of residence	
Total valid	70,747
America	65,758 (92.9 %)
Europe	3216 (4.5 %)
Other	1773 (2.6 %)
Age	
Total valid	29,285
0–10 years	2 (0.0 %)
10–20 years	220 (0.8 %)
20–30 years	7717 (26.4 %)
30–40 years	12,353 (42.2 %)
40–50 years	6204 (21.2 %)
50–60 years	2247 (7.7 %)
60–70 years	492 (1.7 %)
70–80 years	43 (0.1 %)
80–90 years	7 (0.0 %)

Note: Data are frequencies (%).

### 2.4 Statistical analysis

Primary outcomes were step count increase (continuous) and challenge success (binary). We calculated step count increase by subtracting the baseline average daily step count from the average daily step count during the active challenge period. Importantly, after a challenge was failed, steps were still recorded until the final challenge day (unless the participant disconnected their step tracker, stopped wearing their step tracker, or requested to delete their account). We computed the average daily step count during the active challenge period by dividing the total steps taken during a challenge through the number of days the challenge lasted. Challenge success was determined by whether the participant achieved all weekly goals of the challenge. When one weekly goal (consisting of specific daily goals) was missed, a challenge was automatically registered as failed. Due to the large sample size (N = 72,974) of this study, even small effects will become significant (Cumming, 2014). Therefore, we emphasize effect sizes expressed in their original measurement units (instead of only significant tests) and confidence intervals (instead of only point parameters), as suggested by Cumming (2014). To ensure scientific independence from the company that provided us with the research data, we pre-registered the study on Open Science Forum (https://osf.io/d237c). With this pre-registration, the company agreed to publish any findings (including null findings) that would result from our analyses. During the analysis process, we consulted with the company, and made decisions with regards to sample selections that impacted the findings. Whenever a decision was made with regards to sample selections, we decided to add a separate sensitivity check as an appendix, to be as transparent as possible about the impact that this had on the findings. Although not included in formal pre-registration, before data-analysis we decided that daily step count changes of 1000 steps or more would be considered clinically relevant (see *Appendix A* for a rationale). We excluded outliers (see *Appendix B* for rationale) who had a daily average of >60,000 steps (n = 13) or < 1000 steps (n = 841) during the challenge. In *Appendix C* we report a sensitivity check where these outliers are included. Data analysis was done with IBM SPSS statistics for Mac, version 28 (IBM SPSS Statistics for Mac, n.d.). We dealt with missing cases by using pairwise exclusion and used standard *p* < .05 criterium for determining statistical significance.

#### 2.4.1 Hypothesis testing

Hypothesis 1 **Step counts during the challenge will increase compared to baseline.** We performed a two-tailed paired samples *t*-test comparing the baseline historic daily average steps with the daily average steps during a StepBet challenge. We interpret effect size Cohens d ≥ 0.2, ≥0.5, and ≥0.8 as small, moderate, and large, respectively (Cohen, 1988).

Hypothesis 2 **Challenges started as New Year's Resolutions are less successful.** A Chi square test of independence was performed to investigate if the odds of success differ for New Year's resolution challenges (NYRC: started between the 1st and the 14th of January of each year) compared to challenges started during any other period of the year. We interpret effect size Phi (φ) (df = 2) ≥0.07, ≥0.21, ≥0.35 as small, moderate, and large, respectively (Cohen, 1988).

#### 2.4.2 Exploratory analysis

In additional analyses, we explored whether age, gender, historic daily average step count, number of participants per challenge, and bet amount predicted step increases (forced entry multiple linear regression model) and challenge outcome (forced entry binary logistic regression model). Unstandardised b values and odds ratios are used for interpretation.

## 3 Results

### 3.1 Demographics

Of those who provided demographic information, 86.7 % identified as female, with a mean age of 36.47 years old (*SD = 9.40*), and 42.2 % was between 30 and 40 years of age. 92.9 % of participants were from North, South, and Middle America, with the remaining mostly coming from Europe (4.6 %). See Table 1 for more details on the characteristics of the sample.

### 3.2 Hypothesis testing

Hypothesis 1 Step counts during the challenge will increase compared to baseline.

In line with our hypothesis, daily step count was significantly increased during a StepBet challenge, *t* (72,973) = 189.03, *p* = .000, *d* = 0.700, 95 % CI [0.692; 0.708]. Specifically, daily average step counts during a challenge (*M* = 10,197 steps, *SD* = 4162) increased by 2423 steps (*SD* = 3462) (95 % *CI* [2397; 2448]) (31.2 % increase) compared to baseline (*M* = 7774 steps, *SD* = 3112). This is a medium effect size and exceeds the pre-determined threshold for clinical relevance (>1000 steps). See Table 2 for an overview of the descriptive results.

**Table 2:** Descriptive results per challenge outcome (N = 72,974).

	Winner (n = 53,281)	Loser (n = 19,693)	Total (N = 72,974)
Baseline daily step count	7869 (3059)	7561 (3235)	7774 (3112)
Challenge daily step count	11,334 (3661)	7118 (3868)	10,197 (4162)
Change in daily step count	3465 (3013)	−398 (3013)	2423 (3462)
Relative change in step count	+44.0 %	−5.3 %	+31.2 %
Challenge success odds	1	0	0.73

Note: Data are means (SD) and percentages.

Additionally, we explored changes in daily average step counts separately for winners and losers of a StepBet challenge. Firstly, there was a difference in daily step count change between winners and losers, *t* (72,972) = −154.0, *p* = .000, *d* = −1.28, 95 % *CI* [−1.30; −1.27]. Secondly, we explored whether these changes were significant for winners and losers separately. For winners, daily step count was significantly increased during a StepBet challenge, *t* (53,280) = 265.5, *p* = .000, *d* = 1.15, 95 % CI [1.14; 1.16]. Daily average step counts during a challenge increased by 3465 steps (*SD* = 3013) (95 % *CI* [3439; 3490]) (44.0 % increase) compared to baseline. This is a large effect size that exceeds the pre-determined threshold for clinical relevance (>1000 steps). For participants who lost their challenge, daily step count was significantly decreased during a StepBet challenge, *t* (19,692) = −18.64, *p* < .001, *d* = −0.133, 95 % CI [−0.147; −0.119]. Daily average step counts during a challenge decreased by 398 steps (*SD* = 2993) (95 % *CI* [−439; −356]) (5.3 % decrease), compared to baseline. However, this is a small effect size and it does not reach the pre-determined threshold for clinical relevance (>1000 steps).

Hypothesis 2 Challenges started as New Year's Resolutions are less successful.

Cross-tabulation (see Table 3) of challenge success shows that 73.0 % of challenges were successful (winners) and 27.0 % were not successful (losers). In contrast to our hypothesis, a Chi square test of independence showed that challenges started as a New Year's Resolution (start date between the 1st and the 14th of January of each year) have a significantly higher odds of success than challenges started during any other period of the year, *χ2*(1, N = 72,974) = 66.41, *p* ≤ .001, φ = 0.030. Specifically, challenges started as a New Year's Resolution were successful in 77.7 % of the cases, while challenges started during any other period of the year were successful in 72.6 % of the cases. The effect size of this difference is small. Based on these results we conclude that StepBet challenges started as a New Year's Resolution are slightly more likely to be successful.

**Table 3:** Descriptive results of success rates per challenge type (N = 72,974).

	Winner 53,281 (73.0 %)	Loser 19,693 (27.0 %)	Total 72,974 (100 %)
Regular challenge	48,987 (72.6 %)	18,460 (27.4 %)	67,447 (100 %)
New Year's Resolution challenge (start date between 1 and 14 January)	4294 (77.7 %)	1233 (22.3 %)	5527 (100 %)

Note: Data are frequencies and percentages.

### 3.3 Exploratory analysis

We performed exploratory analyses on a subsample of 29,001 participants who did not have missing values for the following variables: gender, age, baseline step count, participants per challenge, and bet amount.

#### 3.3.1 Multiple linear regression model on increase in step count

We combined the independent variables in a model with step count as the dependent variable (see Table 4). No major violations against the assumptions of linearity, multicollinearity, residual variance, and independence were detected. The model explained 4.4 % of the variation in step increase, (*R*^*2*^ *adjusted =* 0.044). All predictors in the model were found to be significant predictors (*p* < .008), but the effects were small. Older age, being a man, and larger games (more participants) were associated with higher step count increases whereas higher baseline step counts and higher bet amounts were associated with lower step count increases. Importantly, the b-values of predictors in this model are too small to predict clinically relevant increases in step counts (>1000 steps).

**Table 4:** Multiple linear regression model on increase in step count (N = 29,002).

	*b*	*95* *% CI for b*	*Beta (ß)*	*t*	*Sig.*
(Constant)	3768.33	3468.83; 4067.83		24.66	<0.001
Gender^a^	−424.50	−541.67; −307.33	−0.041	−7.10	<0.001
Age	33.01	29.16; 36.86	0.097	16.81	<0.001
Baseline step count	−0.188	−0.200; −0.176	−0.181	−31.37	<0.001
Participants per Challenge	0.067	0.018; 0.117	0.015	2.66	0.008
Bet amount	−16.73	−22.43; −11.02	−0.033	−5.75	<0.001

Note: b = unstandardized regression coefficient, B = standardized regression coefficient, 95 % CI for b = Confidence interval for unstandardized regression coefficient.

a Gender: 0 = males, 1 = females.

#### 3.3.2 Multiple binary logistic regression model on challenge outcome

We combined the independent variables in a model with challenge outcome (success/failure) as dependent variable (see Table 5). No major violations against the assumption of linearity between the continuous independent variables and the logit transformation of the dependent variable (Box-Tidwell procedure) were detected. The overall model explained 3.0 % of the variation in challenge outcome, (R^2^ Nagelkerke = 0.030). The model was statistically significant compared to the null model, (χ2(5) = 602.0, *p* < .001), and correctly predicted 73.9 % of challenge outcomes. All variables were found to be significant predictors (p < .001), but the effects were small. Older age, being a man, and larger bet amounts were associated with higher odds of success whereas being female was associated with lower odds of success.

**Table 5:** Multiple binary logistic regression model on challenge outcome (N = 29,002).

	*b*	*Wald*	*Exp(B)*	*95* *% CI for Exp(B)*	*Sig.*
(Constant)	−0.760	46.46	0.468		<0.001
Gender^a^	−0.205	19.94	0.815	0.744; 0.891	<0.001
Age	0.026	283.00	1.026	1.023; 1.029	<0.001
Baseline step count	0.000	106.45	1.000	1.00; 1.00	<0.001
Participants per challenge	0.000	100.85	1.000	1.00; 1.00	<0.001
Bet amount	0.014	46.98	1.014	1.010; 1.018	<0.001

Note: b = unstandardized regression coefficient, B = standardized regression coefficient, 95 % CI for b = Confidence interval for unstandardized regression coefficient.

a Gender: 0 = males, 1 = females.

## 4 Discussion

The aim of this study was to perform a naturalistic evaluation of the effect of participating in a step count challenge with gamified deposit contracts. We found that participating in a StepBet challenge was associated with a 31.2 % increase in step counts compared to baseline. The average challenge success rate was 73 %. Succeeding in a challenge was associated with a large and clinically relevant increase in step counts (44 %), while failing a challenge was related to a slight reduction in step counts (−5.3 %). It is possible that a setback effect after failure caused participants to stop tracking their steps or reduce their efforts in improving their step count. Furthermore, unexpectedly, we found that New Year's resolution challenges were more successful than challenges started during the rest of the year. Several characteristics of challenges and of participants were significant predictors of step counts and challenge success, but were not considered clinically relevant due to low effect size.

In line with our hypothesis, average daily step counts during a challenge increased by 2423 steps (or 31.2 %) compared to baseline. We explain this result through the idea that participating in a gamified deposit contract increases engagement with and effectiveness of a mobile behavior change intervention. This finding is in line with earlier findings on the effects of gamification (Mazeas et al., 2022) and deposit contracts on physical activity (Budworth et al., 2019; Burns and Rothman, 2018; de Buisonjé et al., 2022; Donlin Washington et al., 2016; Krebs and Nyein, 2021; Patel et al., 2016; Stedman-Falls and Dallery, 2020). The size of this effect has mortality reducing potential. For example, research has shown that a 1700 daily steps increase is related to a 41 % reduction in overall mortality among elderly women (Lee et al., 2019). Furthermore, the effect size we found greatly exceeds what is commonly found in randomized controlled trials. Meta-analysis of randomized controlled trials with financial incentives reported an average daily step count increase of 607 steps (10–15 % increase compared to baseline) (Mitchell et al., 2019). However, this meta-analysis included financial incentives that did not require a personal monetary deposit in the form of a deposit contract. It is possible that the deposit contract used in the StepBet challenge further increased effectiveness (compared to regular financial incentives) through exploiting loss aversion. This would be in line with recent meta-analysis by Boonmanunt et al. (2022) who showed that deposit contracts were the most effective type of financial incentive. Yet, caution is warranted when trying to explain these findings. Since the intervention consisted of a combination of gamification elements and deposit contracts, it is impossible to determine which intervention elements specifically were related to this increase in step counts. For example, besides the deposit contract, the intervention also helped participants set concrete (personally tailored) daily step goals, and organized social support by allowing challenge participants to communicate with each other. We know from previous research that goal setting increases physical activity (Mcewan et al., 2015), and that social support is positively related to physical activity (Mendonça et al., 2014). Therefore, although we consider the deposit contract to be the key feature of this intervention, we assume that additional elements such as the gamified microgoals and social support partly explain the effects we found. To determine the isolated effects of the deposit contract element of the Stepbet challenges, future research should compare a StepBet challenge with all active gamification elements but no deposit requirement to a full-fledged StepBet challenge that does have a deposit requirement.

Interestingly, those who succeeded in their challenge increased their daily step count by 3465 steps (or 44 %) while those who failed their challenge decreased their step count by 398 steps (− 5.3 %). Succeeding in a challenge was related to a large and clinically relevant increase in step counts. Failing a challenge was related to slightly lower step counts, although this reduction was not large enough to be considered clinically relevant. Since this study was observational, causal explanations are not possible. Therefore, we have to speculate on what explains this finding. The StepBet challenges are engineered so that after failing one weekly goal, the overall challenge for that person is failed and the monetary deposit is forfeited. Although participants can still track their step counts, and enjoy the gamified elements of the app, this failure (and loss of deposit) might lead to disappointment and demotivation. Perhaps, after a challenge is failed, participants become less motivated to track their step counts (by carrying their smartphone or wearing their external activity tracker) or to actually increase their step counts. In contrast, participants who succeed in their challenge might sustain their efforts until the final challenge day. Although this explanation seems plausible, we cannot rule out the possibility that participants reduce their step count for external reasons and therefore fail their challenge. The dataset we received only contained aggregated data, and not day-by-day step counts. Therefore, we were unable to specifically investigate what happens to daily step counts when a challenge was failed. Future analysis of StepBet data could investigate what happens to step counts when a challenge is failed, and whether this is caused by changes in measurement behavior or in physical activity. Although we cannot ascertain what produced the decrease in step counts we found among losers, it is possible that these participants experienced what has been dubbed a setback effect. In everyday situations of goal striving, Wenzel et al. (2020) have shown that, after an initial instance of failure, a ‘setback effect’ occurs and people are more likely to fail again on subsequent attempts. Others have shown that this effect is related to self-efficacy (ten Broeke and Adriaanse, 2023), and that people can be protected against it by helping them make external attributions (“the weather was just too bad to go outside”), rather than internal attributions (“I am a lazy person”) for their self-regulation failure (Adriaanse and ten Broeke, 2022) Possibly, people who failed a Stepbet challenge made (partly) internal attributions, experienced reduced self-efficacy after failure, and decreased their efforts in goal striving. Future research could develop a simple intervention that helps people in failed Stepbet challenges to make an external attribution and measure their subsequent goal striving to investigate if this protects against the setback effect. Another option to maintain engagement in physical activity when a challenge is failed could be to allow participants to re-enter the challenge with a ‘double or nothing’ option. Research has shown that breaking a streak of successful goal achievement can reduce subsequent goal striving, but this reduction is attenuated when participants are offered the option to repair their streak (Silverman and Barasch, 2022). Future research could investigate the effects of offering a double or nothing option to participants who fail their challenge.

Unexpectedly, challenges started as a New Year's resolution were slightly more successful (77.7 %) than those started during the rest of the year (72.6 %). We hypothesized that New Year's resolution challenges would be less successful than challenges started during other periods of the year, because previous research showed that DietBet challenges for weight loss in January had a lower success rate than during other months (Hirt-Schierbaum and Ivets, 2020). Our results are not in line with this finding, and show that New Year's resolutions for increasing step counts are in fact slightly more successful. Possibly, improving step counts differs from weight loss because it is under direct control of the participant and not a proxy of other behaviors such as eating and physical activity - as is the case for weight loss. Another explanation could be that a resolution to improve step counts is more successful because it is an approach-oriented goal, whereas losing weight is an avoidance-oriented goal. Previous research on New Year's resolution challenges has shown that challenges with approach-oriented goals were more successful (58,9 %) than avoidance-oriented goals (47.1 %) (Oscarsson et al., 2020). Since interest to pursue goals is heightened at the end of the year, and our results show that these goals might be pursued with a higher success rate, future research should study what makes New Year's resolutions for increasing step counts more successful than challenges started during other periods of the year.

Finally, the exploratory regression models for predicting challenge success odds and step count increases only explained a small part of the variance. Although all predictors in both models were significant, none of the predictors had a clinically relevant effect size on challenge success odds or step count increases. Being a man, and being older predicted both slightly higher increases in step counts, and also slightly higher odds of success. It is possible that men and older people respond better to a gamified deposit contract, but it is also possible that, through a selection bias, the men and older people in our sample were more motivated to improve their physical activity. Additionally, being part of a challenge with more participants (and therefore a larger potential prize) predicted a small increase in step counts, but had no effect on odds of success. Furthermore, higher baseline step counts predicted lower step count increases, but had no relationship to the odds of success. Finally, a higher bet amount predicted lower step count increases, but higher odds of success. Although speculative, perhaps a higher bet amount increases the focus on goal achievement, but not on physical activity in itself. For future research, we recommend measuring additional demographic information of participants (e.g., income, educational level), and psychological variables (e.g., motivation, self-efficacy) to further investigate which subgroups benefit most from participating in a StepBet challenge.

### 4.1 Strengths and limitations

An important strength of this study is that we analyzed >70,000 StepBet challenges that were performed over the course of 5 years, whereas most research on deposit contracts reports findings based on small samples (often because low uptake is an obstacle) and limited time frames. Therefore, this study provides a naturalistic evaluation of the true effectiveness (and not mere efficacy) of gamified deposit contracts implemented in real life. This large dataset also allowed us to report effect sizes with tight confidence intervals, which means that we are relatively certain that the effects we found in this sample also exist in the population at large. Finally, participants who started StepBet challenges did this on their own initiative, and this resulted in a demographically heterogenous sample that was not recruited by the researchers. However, this also invited a self-selection bias. We assume that our sample consisted of participants that were motivated to improve their physical activity, were (made) aware of the existence of the StepBet challenges, and were able and willing to make a financial deposit of their own money. Furthermore, an important limitation of this study was the lack of a control condition that was not exposed to the gamification elements or deposit contract. Therefore, we cannot draw causal conclusions on the effect of participating in a StepBet challenge. Instead, we used the available baseline data and determined the within-participant changes in step counts. However, the baseline data was not entirely comparable to the challenge data because the baseline data was trimmed (low and high outliers were excluded before the baseline was determined, see *appendix B* for more detail). Therefore, caution is warranted when drawing conclusions on step count improvements compared to this trimmed baseline. To overcome this limitation, we performed a sensitivity check that only included non-trimmed baselines and report the results in *appendix D*. The pattern of results was not affected in a major way, but step increases among winners were attenuated. Finally, because a StepBet challenge contained all 7 persuasive gamification strategies (including the deposit contract) identified by Cugelman (2013), we cannot ascertain which elements of the challenge produced the effects.

### 4.2 Implications

Randomized controlled trials already identified gamified deposit contracts as an effective tool to support health behavior change. The current findings add to the existing evidence base by showing that, in real world conditions, among a large and diverse sample, gamified deposit contracts are associated with clinically relevant increases in physical activity. Although our study design does not allow for causal explanations, it appears plausible that participating in a gamified deposit contract challenge helped participants increase their physical activity. The effects we found provide further support for implementing (elements of) gamified deposit contracts to improve physical activity in future behavior change interventions. Furthermore, our findings show that New Year's resolution challenges are more effective than other challenges. Therefore, we suggest that StepBet (and other intervention providers) stimulate their participants to make use of New Year's resolution challenges and increase their odds of successful behavior change. Finally, because it is unknown how acceptable gamified deposit contracts are among people with cardiovascular disease or other chronic conditions, future research should explore whether these more vulnerable subgroups might also benefit from this type of intervention. Ultimately, we hope that the current work will help inform public health policy making and may inspire future intervention design of behavior change interventions that improve population health.

### 4.3 Conclusion

In a real-world setting, and among a large and diverse sample, participating in a physical activity challenge using gamified deposit contracts was associated with a large increase in step counts. We recommend intervention providers to implement gamified deposit contracts for physical activity. However, we urge for more research into potential setback effects (and how to mitigate them) among those who fail their challenge. Finally, New Year's resolution challenges were more effective than regular challenges so we advise to make use of this temporal landmark to increase the odds of successful behavior change.

## CRediT authorship contribution statement

Study design (DB, FB, TR, LB); data acquisition (DB, FB); data analysis and interpretation (DB, FB, AE, EB, LB); drafting the manuscript (DB, FB, HK); manuscript revision (DB, FB, LB, EB, TR, VJ, RK, HK, AE). All authors gave final approval and agreed to be accountable for all aspects of the work ensuring integrity and accuracy.

## Declaration of competing interest

Erika Litvin Bloom is employed as Lead Scientist by WayBetter Inc. and receives salary support and stock options from the company.

## Data Availability

The data that support the findings of this study are available from the corresponding author, DB, upon reasonable request.

## Appendix A Supplementary data

Supplementary material Image 1
