# Supplementary material for: Put your money where your feet are: The real-world effects of StepBet gamified deposit contracts for physical activity
Source: Internet Interv. 2023 Feb 26;31:100610. doi: 10.1016/j.invent.2023.100610 (PMC9982638; doi:10.1016/j.invent.2023.100610)
Supplement: Supplementary file 1 — Supplementary material [file mmc1.docx]

1. **Appendices**

**Appendix A: effect size rationale**

Before data analysis, we determined what would be considered a clinically relevant increase in step counts. Meta-analysis shows that financial incentive interventions increase daily step counts by 10-15% or by 870-2,551 steps per day (Mitchell et al., 2019). A typical challenge in the StepBet app aims for an overall increase in daily step counts of 4 active days (110 % of baseline steps), 2 power days (130% of baseline steps) and 1 rest day. Research shows that a 2,500 daily steps increase is related to a reduction in overall mortality by 41% in elderly women (Lee et al., 2019) and increasing from 4,000 to 8,000 steps caused a decrease in overall mortality by 51% in adults over 40 (Saint-Maurice et al., 2020). Based on the above, we decided that an increase of 1,000 steps or more on a daily basis can be realistically expected and will be considered clinically relevant.

**Appendix B: outlier rationale**

We determined which datapoints were considered outliers based on the following rationale.

StepBet retrieves 90 days of historic step counts at the start of a challenge to tailor intervention goals. During this process, the goal setting algorithm trims low (<1,000 steps/day) and high (>60,000 steps/day) days to calculate a historic average and determine the challenge goals on that trimmed average. However, we did not have day by day data of participants’ step counts during the challenge (we only had their total step count, which included all days; low and high days were not trimmed). Therefore, to make the challenge average more comparable to the historic average, we decided to remove participants whose average daily step count during the challenge was less than 1,000 daily steps or higher than 60,000 daily steps. Based on this cut-off rule, we excluded participants who had a daily average of >60,000 steps (n = 13) or <1,000 steps (n = 841) during the challenge. In appendix C we report a sensitivity check where these outliers are included. Furthermore, in appendix D we report a sensitivity check during which we only analyse cases for which the historic average was not trimmed.

**Appendix C: Analysis with outliers included**

**Figure 1B** – Flowchart of data cleaning process including outliers


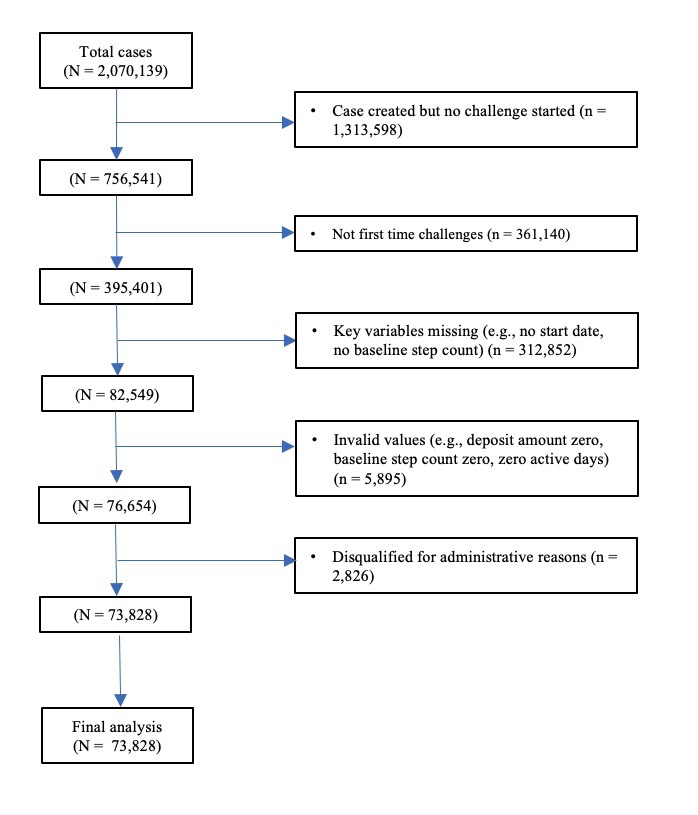


**Table 2B.** Descriptive results per challenge outcome (N = 73,828)

|  | Winner  (N = 53,294) | Loser  (N = 20,534) | Total  (N = 73,828) |
| --- | --- | --- | --- |
| Baseline daily step count | 7,872 (3070) | 7,393 (3261) | 7,739 (3131) |
| Challenge daily step count | 11,350 (3813) | 6,851 (4004) | 10,099 (4361) |
| Change in daily step count | 3,479 (3162) | -543 (3055) | 2,360 (3614) |
| Relative change in step count | +44.2% | -7.3% | +30.5% |
| Challenge success odds | 1 | 0 | .72 |

Note: Data are means (SD) and percentages

**Table 3B.** Descriptive results of success rates per challenge type (N = 72,974)

|  | Winner  53,285 (72.2%) | Loser  20,534 (28.8%) | Total  N = 73,828 (100%) |
| --- | --- | --- | --- |
| Regular challenge | 48,999 (71.8%) | 19,254 (28.2%) | 68,253 (100%) |
| New Year’s Resolution challenge (start date between 1-14 January) | 4,295 (77%) | 1,280 (23%) | 5,575 (100%) |

Note: Data are means (SD) and percentages

**Description of results**

The pattern of results was not affected by including outliers, although the descriptive results changed slightly. This analysis with outliers included shows that step increases among winners was largely unaffected (+44 % vs + 44.2%). However, step reductions among losers increased from -5.3% (398 steps) to -7.3% (543 steps). This can be explained by the fact that most outliers (n = 841) were excluded on the low end of the distribution.

**Appendix D: Analysis with cases who did not have trimmed baseline step counts**

**Figure 1C** – Flowchart of data cleaning process without trimmed baseline step counts


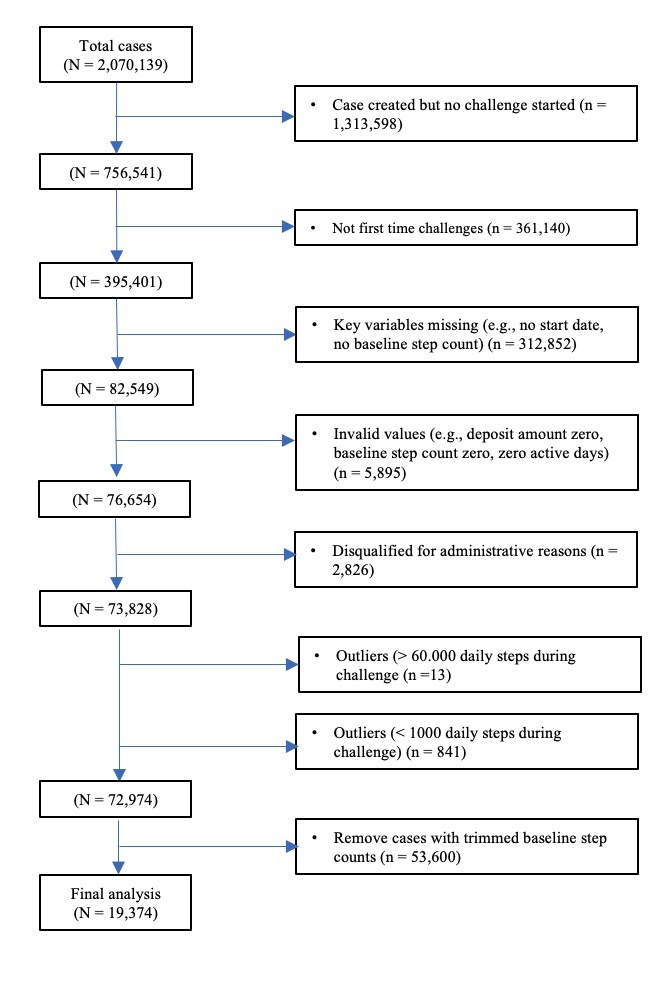


**Table 2C.** Descriptive results per challenge outcome (N = 19,374)

|  | Winner  (N = 14,908) | Loser  (N = 4,466) | Total  (N = 19,374) |
| --- | --- | --- | --- |
| Baseline daily step count | 9,326 (3,007) | 9,250 (3,248) | 9,308 (3,064) |
| Challenge daily step count | 12,302 (3,655) | 88,73 (4,158) | 11,511 (4,044) |
| Change in daily step count | 2,976 (2,537) | -377 (3,029) | 2,203 (3,010) |
| Relative change in step count | +31.9% | -4.8% | +23.7% |
| Challenge success odds | 1 | 0 | .77 |

Note: Data are means (SD) and percentages

**Table 3C.** Descriptive results of success rates per challenge type (N = 72,974)

|  | Winner  14,908 (77.0%) | Loser  4,466 (23.0%) | Total  N = 19,374 (100%) |
| --- | --- | --- | --- |
| Regular challenge | 13,760 (76.7%) | 4,184 (23.3%) | 17,944 (100%) |
| New Year’s Resolution challenge (start date between 1-14 January) | 1,148 (80.3%) | 282 (19.7%) | 1,430 (100%) |

Note: Data are means (SD) and percentages

**Description of results**

The pattern of results was not affected by only analysing cases with non-trimmed baseline step counts, although the descriptive results changed. Baseline step counts for all participants were increased. This can be explained by the fact that the goal setting algorithm mainly cut-off outliers on the low end of the distribution. Furthermore, this analysis shows that step increases among winners decreased (+44 % vs + 31.9%) while step reductions among losers also decreased slightly from -5.3% (398 steps) to -4.8% (377 steps). Finally, this analysis shows that the overall success rate of challenges increased from 73% to 77%. It appears that the goal setting algorithm (by cutting off outliers on the low end of the distribution of baseline step counts) increases baselines (and resulting intervention goals) and therefore makes it harder for participant to succeed in their challenge.
